# Curcumin Supplementation and Human Disease: A Scoping Review of Clinical Trials

**DOI:** 10.3390/ijms24054476

**Published:** 2023-02-24

**Authors:** Timothy M. Panknin, Carol L. Howe, Meg Hauer, Bhanu Bucchireddigari, Anthony M. Rossi, Janet L. Funk

**Affiliations:** 1 College of Medicine, University of Arizona, Tucson, AZ 85724, USA; 2 The University of Arizona Health Science Library, Tucson, AZ 85724, USA; 3 University of Arizona Cancer Center, University of Arizona, Tucson, AZ 85724, USA; 4 Department of Physiology, Honors College, University of Arizona, Tucson, AZ 85724, USA; 5 Department of Medicine and School of Nutritional Sciences and Wellness, University of Arizona, Tucson, AZ 85724, USA

**Keywords:** curcumin, curcuminoids, turmeric, *Curcuma longa* L., human clinical trials, dietary supplement

## Abstract

Medicinal properties of turmeric (*Curcuma longa* L.), a plant used for centuries as an anti-inflammatory, are attributed to its polyphenolic curcuminoids, where curcumin predominates. Although “curcumin” supplements are a top-selling botanical with promising pre-clinical effects, questions remain regarding biological activity in humans. To address this, a scoping review was conducted to assess human clinical trials reporting oral curcumin effects on disease outcomes. Eight databases were searched using established guidelines, yielding 389 citations (from 9528 initial) that met inclusion criteria. Half focused on obesity-associated metabolic disorders (29%) or musculoskeletal disorders (17%), where inflammation is a key driver, and beneficial effects on clinical outcomes and/or biomarkers were reported for most citations (75%) in studies that were primarily double-blind, randomized, and placebo-controlled trials (77%, D-RCT). Citations for the next most studied disease categories (neurocognitive [11%] or gastrointestinal disorders [10%], or cancer [9%]), were far fewer in number and yielded mixed results depending on study quality and condition studied. Although additional research is needed, including systematic evaluation of diverse curcumin formulations and doses in larger D-RCT studies, the preponderance of current evidence for several highly studied diseases (e.g., metabolic syndrome, osteoarthritis), which are also clinically common, are suggestive of clinical benefits.

## 1. Introduction

**Figure 1:**
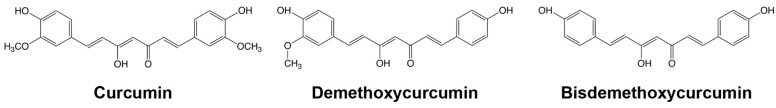
Chemical structures of turmeric-derived curcuminoids, of which curcumin is the most abundant.

Turmeric, derived from the dried rhizome of *Curcuma longa* L., a tropical plant native to India and southeast Asia, has been used for centuries as a spice, dye, and medicine [1]. Although multiple turmeric constituents have differential in vivo physiological effects in pre-clinical models when administered in isolation or in chemically complex turmeric extracts of variable composition [2], current interest in the medicinal turmeric properties has primarily focused on its structurally related polyphenols (curcuminoids), of which curcumin is the primary constituent (Figure 1) [3,4]. Curcuminoids comprise 3% by weight of dried turmeric rhizome and are the source of ground turmeric rhizome’s orange hue [3]. Medicinal use of turmeric has its origins in Ayurvedic medicine with a clear history of continuous use since around 500 BCE, with additional evidence suggesting its possible medicinal use since 2500 BCE, which would extend turmeric’s period of medicinal use to 4500 years [1,5].

More recently, turmeric has been adopted into Western medicinal practices. Curcuminoid-enriched turmeric supplements have been promoted in the lay press to treat various ailments from osteoarthritis to cancer. In recent decades in the United States (US), sales of minimally regulated dietary supplements are part of a large and growing global nutraceutical business [6], with botanical supplements alone bringing in USD 12.4 billion in US sales in 2021 with an annual growth rate exceeding USD 1 billion [7]. Turmeric dietary supplement use has grown in recent years as part of this trend, becoming a top-selling botanical dietary supplement in the United States [7,8]. Based on these figures and the popularity of curcumin use, it appears that people are looking for affordable, natural products to improve their lives and cure their ailments. Indeed, in recent years, use of turmeric dietary supplements, which in the US are primarily formulated to contain curcuminoid-enriched (98%) extracts [4], has been documented in epidemiologic studies by one-third of those with rheumatoid arthritis [9] and almost one-quarter of women diagnosed with breast cancer [10].

In both in vitro and/or in vivo pre-clinical studies, among other beneficial effects, curcumin has shown promise in ameliorating inflammation associated with chronic disease or infection, and limiting cancer proliferation and progression [2,11,12,13,14]. Questions remain as to whether these benefits extend to humans [15,16]. For example, despite evidence of in vivo bioactivity in rodent models, there have been concerns about curcuminoid bioavailability since curcuminoids undergo hepatic conjugation and primarily circulate as inactive glucuronides when ingested by humans (and rodents), a fate shared with many dietary polyphenols [2,17,18]. Emerging evidence, however, suggests that bioactive curcumin can be reformed in vivo from circulating curcumin glucuronides via enzymatic deconjugation [17,18]. The multiplicity of defined targets for curcuminoid action has also been a topic of concern [15], although in vivo metabolism may also provide an explanation here [2]. For example, certain oxidative curcumin metabolites have been demonstrated to form covalent adducts with specific proteins, including the proinflammatory transcription factor NFκB [19,20,21,22,23], a pharmacologic strategy also successfully employed for several FDA-approved drugs [24,25]. However, this multiplicity of action has also led curcuminoids to be labeled as “PAINS” (pan-assay interference compounds) or “IMPS” (invalid metabolic panaceas) by researchers who have additionally claimed without supporting evidence that “no double-blinded, placebo-controlled clinical trial of curcumin has been successful” [15].

To determine what level of evidence for the medicinal effects of curcuminoids (to be referred to here as curcumin) exists in human clinical trials, a scoping review of the literature was conducted. In contrast with systematic reviews, which are designed to answer narrower questions and are limited to specific study types, a scoping review methodology was chosen in order to build a comprehensive overview of the topic, identify existing evidence, and expose gaps in research [26,27]. To this end, various study designs and publication types were included in this scoping review of studies of orally administered curcumin-containing products targeted for disease treatment [26,27].

## 2. Results

### 2.1. Identification of Relevant Citations

**Figure 2:**
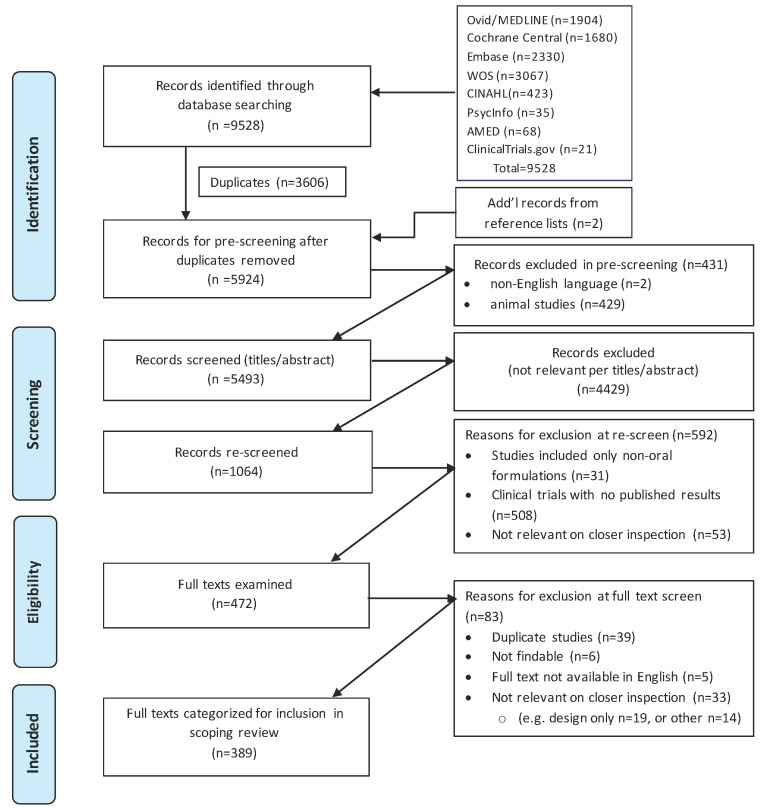
Preferred Reporting Items for Systematic Reviews (PRISMA) flow diagram of scoping review process used to search literature and extract citations meeting inclusion criteria.

Eight databases were systematically searched as described in the Methods section using PRISMA Extension for Scoping Reviews (PRISMA-ScR) guidelines [28], yielding 9528 citations for clinical trials testing oral administration of curcumin-containing products for disease treatment (Figure 2). After removal of 3606 duplicates, 429 animal studies, and two non-English language studies, and addition of two citations identified in references, two reviewers independently screened the remaining 5924 records. Of these citations, 4429 were excluded at the title and abstract level because of irrelevance to the topic. Titles and abstracts were rescreened, and a further 592 were excluded for irrelevance (e.g., non-oral formulations, report of clinical trial designs without data). After review of the full texts of the remaining 472 citations, the 389 citations found to meet all criteria were categorized according to disease/condition targeted with data related to trial design and findings extracted, collated, and summarized.

### 2.2. Types and Trends in Conditions Studied

**Figure 3:**
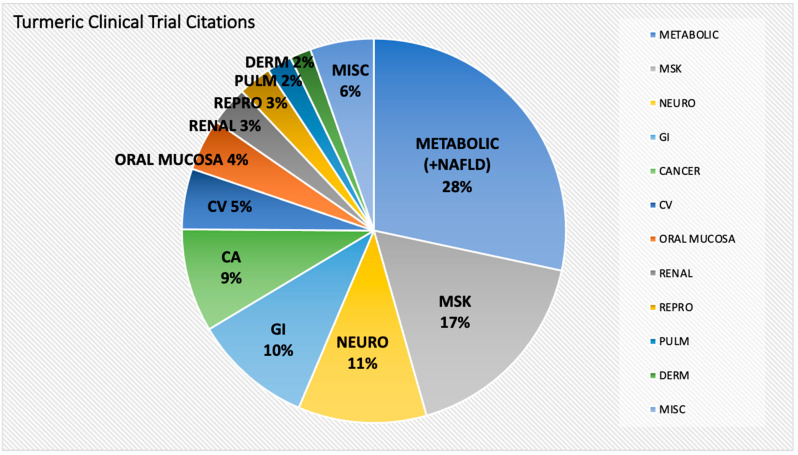
Turmeric clinical trials by organ system or disease process. Clinical trials were organized for analysis by organ system (e.g., musculoskeletal [MSK], neuropsychiatric [NEURO], gastrointestinal [GI], cardiovascular [CV], oral mucosa, renal, reproductive organs [REPRO], pulmonary [PULM], or dermatologic [DERM] disorders) or disease process (metabolic disorders including non-alcoholic fatty liver disease [METABOLIC + NAFLD] or cancer [CA]).

Of the disease processes studied, curcumin clinical trials related to the treatment of metabolic abnormalities associated with obesity and insulin resistance were the most prevalent (22%) [29,30,31,32,33,34,35,36,37,38,39,40,41,42,43,44,45,46,47,48,49,50,51,52,53,54,55,56,57,58,59,60,61,62,63,64,65,66,67,68,69,70,71,72,73,74,75,76,77,78,79,80,81,82,83,84,85,86,87,88,89,90,91,92,93,94,95,96,97,98,99,100,101,102,103,104,105,106,107,108,109,110,111,112,113,114], including treatment of hyperglycemia and/or insulin resistance, hyperlipidemia, hypertension, and obesity-associated inflammation. When inclusive of citations examining non-alcoholic fatty liver disease (NAFLD) [115,116,117,118,119,120,121,122,123,124,125,126,127,128,129,130,131,132,133,134,135,136,137,138], a hepatic manifestation of metabolic syndrome [139], clinical trial citations focused on metabolic disorders (METABOLIC) accounted for almost one-third of curcumin clinical trial citations (Figure 3). Musculoskeletal (MSK) disorders were the second most common diseases targeted (17%) [140,141,142,143,144,145,146,147,148,149,150,151,152,153,154,155,156,157,158,159,160,161,162,163,164,165,166,167,168,169,170,171,172,173,174,175,176,177,178,179,180,181,182,183,184,185,186,187,188,189,190,191,192,193,194,195,196,197,198,199,200,201,202,203,204,205,206], followed by neurologic conditions (NEURO, 11%) [207,208,209,210,211,212,213,214,215,216,217,218,219,220,221,222,223,224,225,226,227,228,229,230,231,232,233,234,235,236,237,238,239,240,241,242,243,244,245,246,247,248], gastrointestinal diseases (GI, excluding NAFLD) (10%) [249,250,251,252,253,254,255,256,257,258,259,260,261,262,263,264,265,266,267,268,269,270,271,272,273,274,275,276,277,278,279,280,281,282,283,284,285,286,287], and cancer (CA, 9%) [288,289,290,291,292,293,294,295,296,297,298,299,300,301,302,303,304,305,306,307,308,309,310,311,312,313,314,315,316,317,318,319,320,321]. Together, these top five disease categories accounted for 75% of curcumin clinical trial citations. Less studied diseases or organ systems in curcumin clinical trials included the cardiovascular system (CV, 5%) [322,323,324,325,326,327,328,329,330,331,332,333,334,335,336,337,338,339,340,341], oral mucosa (4%) [342,343,344,345,346,347,348,349,350,351,352,353,354,355,356,357,358], kidney (RENAL, 3%) [359,360,361,362,363,364,365,366,367,368,369,370,371], reproductive organs (REPRO, 3%) [372,373,374,375,376,377,378,379,380,381,382], lungs (PULM, 2%) [383,384,385,386,387,388,389,390], skin (DERM, 2%) [391,392,393,394,395,396,397], or other miscellaneous disease processes (MISC, 6%) [398,399,400,401,402,403,404,405,406,407,408,409,410,411,412,413,414,415,416,417,418].

**Figure 4:**
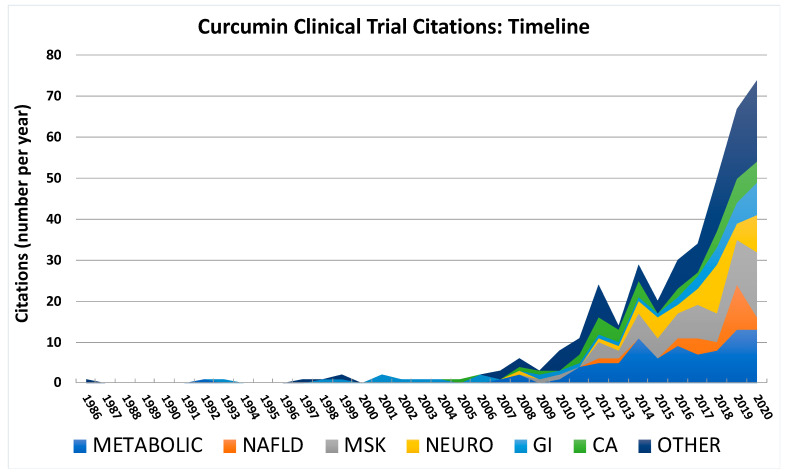
Publication timeline for curcumin clinical trials. Citations per year are presented in stacked plots to demonstrate secular trends for the five most common categories, including metabolic (with NAFLD graphed separately), musculoskeletal (MSK), neuropsychiatric (NEURO), and gastrointestinal (GI, excluding NAFLD) disorders or cancer [CA]. All other diseases (OTHER) are graphed as a group.

When examining trends in diseases studied over time (Figure 4), a small number (n = 15) of turmeric clinical trial citations primarily related to the treatment of gastrointestinal disorders (n = 10) appeared sporadically over a 20 year period following an initial 1986 report examining effects on post-operative inflammation [417]. However, after this period, a notable secular change occurred as curcumin clinical trial citations during the last two decades increased almost exponentially. Citations for some of the most studied disease categories reflected this dramatic rise (e.g., metabolic [with or without NAFLD] or musculoskeletal disorders), while for other disease categories, such as cancer, citation increases were more modest. Diseases that were initially a primary focus of study (e.g., gastrointestinal disorders, excluding NAFLD) were no longer the most common, while other conditions were only a focus of study within the last ten years (e.g., pulmonary, reproductive, and renal diseases), including a marked increase in citations reporting beneficial effects in NAFLD, a disease process first described 20 years ago [139].

### 2.3. Measures of Curcumin Clinical Trial Quality

**Figure 5:**
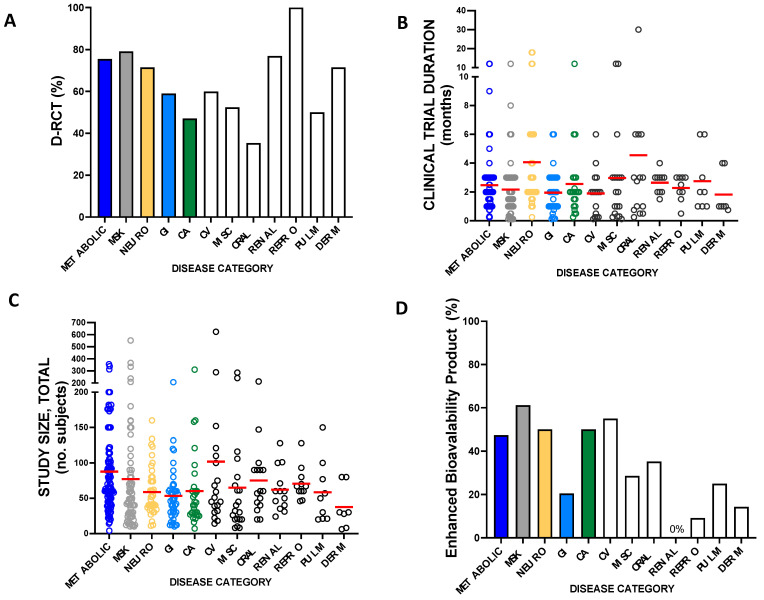
Curcumin clinical trial design elements by disease category. (**A**) Prevalence of citations reporting double-blind, randomized, placebo-controlled (D-RCT) trial results. (**B**) Curcumin clinical trial duration or (**C**) size, noting individual citations (open circles) and averages (red line). (**D**) Prevalence of citations reporting enhanced bioavailability curcuminoid product treatment effects.

While assessment of study quality is not an obligatory aspect of a scoping review, several key clinical trial design features were examined. Most important among these was an analysis of the prevalence of citations reporting results from double-blind, randomized placebo-controlled trials (D-RCT), a gold standard design for clinical trials [419], albeit one that tends to minimize treatment effects [420]. A D-RCT design was utilized in 70% of citations reporting curcumin clinical trial results. Amongst the top five diseases studied, a D-RCT design was most common for musculoskeletal disorders (79.1%) and least common for cancer (47.1%) (Figure 5A). Curcumin clinical trial duration was also assessed and ranged from 4 days to 30 months (Figure 5B) with an average (+/−SD) duration of 2.6 +/− 2.8 months, and median duration of 2.0 months. Trials studying neurologic disorders tended to be of longer duration (4.1 ± 4.1 months) as compared to trials for other conditions, such as metabolic disease (2.5 ± 1.9 months, *p* < 0.05) or musculoskeletal disorders (2.2 ± 2.0 months, *p* < 0.05). While the statistical power of clinical trials was not assessed in this scoping review, curcumin clinical trial study sizes were examined (Figure 5C), varying from a low of n = 4 subjects (treatment arm, n = 2) to a high of n = 624 (treatment arm, n = 313), with an average study size of n = 73 ± 68 (treatment arms, n = 35 ± 33) and a median size of n = 58 (treatment arms, n = 35). Average cohort sizes were similar across disease states, but among the most studied conditions, tended to be largest for metabolic disease (median, n = 65; range n = 4–358), followed by musculoskeletal disorders (median, n = 49; range n = 10–552).

Almost all curcumin trials assessed treatment effects of curcumin products formulated to contain turmeric-derived curcuminoid-enriched extracts that are broadly analogous to most turmeric supplements sold in the United States [4]. Due to concerns about curcumin bioavailability, a large share (55%) of the US turmeric dietary supplement market is comprised of products formulated to enhance curcumin bioavailability, including proprietary products where curcuminoid extracts are often combined with some type of lipophilic carrier to increase absorption, or products combining curcumin with piperine to decrease metabolism [4]. The proportion of curcumin clinical trials testing enhanced bioavailability curcumin products was therefore also evaluated (Figure 5D). Overall, 45% of curcumin clinical trials assessed enhanced bioavailability curcumin products. Among the most commonly studied diseases, enhanced bioavailability curcumin products were most studied for musculoskeletal disorders (61.2%), while gastrointestinal disorders (GI) were among the lowest (20.5%), likely due to intestinal (not systemic) targeting in most (69%) GI studies. The prevalent use of curcumin products with improved bioavailability impedes meaningful comparison of curcuminoid doses tested across studies. This is due to the variable effects of these products on curcumin bioavailability, which are rarely evaluated within the context of clinical trials. Because a meaningful comparison of curcuminoid dosing across citations was therefore not possible, dosing information was not analyzed.

### 2.4. Side Effects Reported in Curcumin Clinical Trial Citations

The most frequently reported side effects associated with curcumin included GI symptoms (diarrhea, abdominal pain, flatulence, yellow stools, dyspepsia, nausea, vomiting, GI distress, constipation), headache, and dizziness. Most were classified as mild. Serious side effects were uncommon but included a single report of worsening cachexia and muscle wasting in a pancreatic cancer trial, resulting in increased morbidity and mortality [313], as well as an increased incidence of acute kidney injury with perioperative curcumin treatment when undergoing elective abdominal aortic aneurysm repair [336]. Uncommon side effects included hair loss, mild fever, and throat infection.

### 2.5. Clinical Trials for Metabolic Disorders

**Figure 6:**
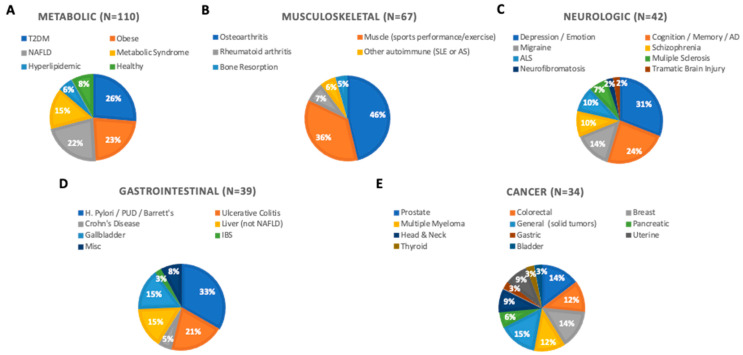
Frequency of specific conditions studied within each of the top five categories, which together accounted for 75% of curcumin clinical trial citations. N = number of citations.

Clinical trial citations reporting curcumin-associated effects on disordered glucose and lipid metabolism, including those focused on NALFD, represented almost one-third of curcumin clinical trial citations [29,30,31,32,33,34,35,36,37,38,39,40,41,42,43,44,45,46,47,48,49,50,51,52,53,54,55,56,57,58,59,60,61,62,63,64,65,66,67,68,69,70,71,72,73,74,75,76,77,78,79,80,81,82,83,84,85,86,87,88,89,90,91,92,93,94,95,96,97,98,99,100,101,102,103,104,105,106,107,108,109,110,111,112,113,114,115,116,117,118,119,120,121,122,123,124,125,126,127,128,129,130,131,132,133,134,135,136,137,138]. Most of these studies utilized a D-RCT design (76%), and 48% focused on enhanced bioavailability curcumin products. The studies evaluating metabolic disorders included relatively large cohorts (mean, n = 88), and had an average study duration of 2.5 months. In study populations described as healthy or hyperlipidemic [29,30,31,32,33,34,35,36,37,38,39,40,41,42,43], representing only 14% of citations in this category (Figure 6A), beneficial effects of curcumin on lipid or glucose metabolism were uncommon. In contrast, the majority of citations for studies evaluating the metabolic effects of curcumin in insulin-resistant populations with obesity (26% of studies in this category) [44,45,46,47,48,49,50,51,52,53,54,55,56,57,58,59,60,61,62,63,64,65,66,67,68], metabolic syndrome (15%) [69,70,71,72,73,74,75,76,77,78,79,80,81,82,83,84,85], or type 2 diabetes mellitus (26%) [86,87,88,89,90,91,92,93,94,95,96,97,98,99,100,101,102,103,104,105,106,107,108,109,110,111,112,113,114] reported positive outcomes for commonly studied endpoints. These endpoints included lipids (72%), glucose and/or HbA_1c_ levels (82%), measures of insulin resistance (92%), biomarkers of inflammation (61%) or oxidative stress (69%), and/or improvements in weight/BMI (73%). The majority of citations for clinical trials in populations with NAFLD (a hepatic manifestation of metabolic syndrome) populations (23% of studies in this category) [115,116,117,118,119,120,121,122,123,124,125,126,127,128,129,130,131,132,133,134,135,136,137,138], also reported beneficial outcomes for glucose (83%) and lipid (75%) metabolism. Improved liver function (n = 11 of 16 studies where this was examined) and/or liver steatosis or fibrosis (n = 5 of 6 studies) were also reported.

### 2.6. Clinical Trials for Musculoskeletal Disorders

Disorders of the musculoskeletal system were the second most studied category (17% of total) [140,141,142,143,144,145,146,147,148,149,150,151,152,153,154,155,156,157,158,159,160,161,162,163,164,165,166,167,168,169,170,171,172,173,174,175,176,177,178,179,180,181,182,183,184,185,186,187,188,189,190,191,192,193,194,195,196,197,198,199,200,201,202,203,204,205,206]. Most of these citations reported D-RCT results (79%) of enhanced bioavailability products (61%) and had an average study duration of 2.2 months. The most common MSK disorder studied was osteoarthritis (46%) (Figure 6B) [140,141,142,143,144,145,146,147,148,149,150,151,152,153,154,155,156,157,158,159,160,161,162,163,164,165,166,167,168,169,170], a common age-related joint disorder for which obesity, as with metabolic dysfunction, is a risk factor. Muscle outcomes related to sports performance or exercise (e.g., soreness) comprised the second most common musculoskeletal condition studied (36%) [171,172,173,174,175,176,177,178,179,180,181,182,183,184,185,186,187,188,189,190,191,192,193,194]. The remaining 18% of MSK citations reported trial results for miscellaneous disorders, including autoimmune rheumatic disorders (rheumatoid arthritis [n = 5 citations] [195,196,197,198,199], systemic lupus erythematosus [SLE, n = 3] [200,201,202], ankylosing spondylitis [AS, n = 1] [203]) or osteoporosis (n = 3) [204,205,206], including menopause-related bone loss where bone protective effects were reported [205]. Most osteoarthritis trials reported clinical outcomes [140,141,142,143,144,145,146,147,148,149,150,151,152,153,154,155,156,157,158,159,160,161,162,163,164,165,166,167,168,169,170], with approximately half also reporting treatment effects on various biomarkers. Most clinical outcomes, including pain (92%) and function (86%), as well as biomarkers of inflammation, oxidative stress, and cartilage degradation (80%), showed positive effects or equivalency to non-steroidal anti-inflammatory drugs (NSAIDs). The results also indicated a reduced need for rescue medications. In trials examining curcumin effects on exercise-related changes in muscle [171,172,173,174,175,176,177,178,179,180,181,182,183,184,185,186,187,188,189,190,191,192,193,194], the second most studied MSK condition (n = 24), at least half of the citations provided data for each of several different clinical and/or biomarker outcomes. Most studies reported beneficial effects on pain (82%) or functional outcomes (75%), as well as reductions in creatinine kinase levels (58%), a measure of muscle damage, and/or beneficial effects on other biomarkers (e.g., inflammation or oxidative stress) (56%). The third most studied musculoskeletal condition (7%, n = 5 citations), rheumatoid arthritis, is an autoimmune disorder distinct from osteoarthritis. Here, results were mixed [195,196,197,198,199], with only two D-RCTs out of four RA studies (of which n = 3 were D-RCTs) demonstrating improved clinical outcomes as well as significant improvements in biomarkers of inflammation [195,197]. 

### 2.7. Clinical Trials for Neuropsychiatric Disorders

Neuropsychiatric disorders comprised the third most commonly studied category of disorders (11% of citations, n = 42) (Figure 6C) [207,208,209,210,211,212,213,214,215,216,217,221,222,223,224,225,226,227,228,229,230,231,232,233,234,235,236,237,238,239,240,241,242,243,244,245,246,247,248], with most citations reporting results from D-RCT studies (71%). Studies were of longer duration (4.1 months) compared to most other top categories (*p* < 0.05), with half testing enhanced bioavailability products (50%). While a range of conditions was studied (e.g., migraine [n = 6 citations reporting unique data from two studies] [207,208,209,210,211,212], schizophrenia [n = 4] [213,214,215,216], amyotrophic lateral sclerosis [ALS, n = 4] [217,218,219,220], multiple sclerosis [MS, n = 3] [221,222,223], neurofibromatosis [n = 1] [224], or traumatic brain injury n = 1] [225]), more than half of the citations focused either on depression (n = 13, 31%) [226,227,228,229,230,231,232,233,234,235,236,237,238] or cognition (n = 10, 24%) [239,240,241,242,243,244,245,246,247,248]. Clinical trial citations reporting curcumin effects on depression in populations with or without a major depressive disorder were numerous [226,227,228,229,230,231,232,233,234,235,236,237,238] but with mixed results; an absence of effect was noted in half of the reports, while in other citations differences were reported with respect to ameliorating depression vs. anxiety. For clinical trials related to cognition, two D-RCTs conducted over a decade ago reported negative results in probable Alzheimer’s disease populations when examining non-bioenhanced curcumin in 6-month-long studies [247,248]. These were followed in the last decade by seven D-RCT that were larger (n = 20–50 per arm vs. n = 10 in AD) and tested enhanced bioavailability products in a different population, aged adults without dementia or AD [239,240,241,242,243,244,246]. Benefits were reported in all but one [244] of these later cognition studies, which also used more detailed assessments of cognition. Additionally, two studies examining positron emission tomography (PET) imaging of brain plaques also reported improvements [243,246].

### 2.8. Clinical Trials for Gastrointestinal Disorders (Excluding Cancer)

Clinical trials examining curcumin effects on gastrointestinal (GI) disorders [249,250,251,252,253,254,255,256,257,258,259,260,261,262,263,264,265,266,267,268,269,270,271,272,273,274,275,276,277,278,279,280,281,282,283,284,285,286,287], a primary focus of early curcumin trials more than twenty years ago, represented only 10% of citations when excluding NAFLD (Figure 6D). The percentage of GI citations reporting D-RCT results (59%) was lower than in previously discussed categories. Testing of enhanced bioavailability products was also less common (21%). Most studies focused on the intestines (69%, n = 27), including disorders of the foregut (n = 13) [249,250,251,252,253,254,255,256,257,258,259,260,261], small intestine (n = 4) [262,263,264,265], and colon (n = 10) [266,267,268,269,270,271,272,273,274,275]; additional citations focused on gallbladder (n = 6) [276,277,278,279,280,281] and liver (n = 6) [282,283,284,285,286,287]. When considered by disease pathogenesis, foregut conditions related to disordered gastric secretion, including peptic ulcer disease and Barrett’s esophagitis, were the most frequently studied (33%, n = 13) [249,250,251,252,253,254,255,256,257,258,259,260,261], followed by inflammatory bowel disease (IBD; 26%, n = 10), including Crohn’s disease or ulcerative colitis [262,263,268,269,270,271,272,273,274,275]. Studies evaluating curcumin effects on peptic ulcer disease or Barrett’s esophagitis were among some of the earliest trials and have continued to the present; however, these trials have yielded mixed results with little evidence of improved symptoms and mixed reports on healing and/or reduction of *H. pylori* infections [249,250,251,252,253,254,255,256,257,258,259,260,261]. While less numerous, results from clinical trials evaluating curcumin effects on IBD were more consistent. Almost two decades ago, the first trials related to IBD appeared; a D-RCT ulcerative colitis (UC) trial reported a beneficial effect of curcumin [270] while no effect was seen in a D-RCT study of a mixed population with UC or Crohn’s disease [268]. With the exception of one study [271], subsequent UC trials over the last decade (n = 6) have consistently reported reductions in clinical symptoms as well as endoscopic improvement and/or decreases in calprotectin or other disease activity biomarkers [268,269,270,271,272,273,274,275]. Curcumin effects on IBD affecting the small bowel (Crohn’s disease) were less clear with benefits reported in only one of two recent D-RCT trials [262,263]. Citations reporting curcumin effects on disorders of the oral mucosa (n = 17) were grouped separately from gastrointestinal disorders (oral mucosa, Figure 3) and primarily focused on gingivitis, canker sores, oral lichen planus, or submucosal fibrosis, reporting benefits on variable endpoints. However, only 35% of these oral mucosa studies were D-RCT [342,343,344,345,346,347,348,349,350,351,352,353,354,355,356,357,358].

### 2.9. Clinical Trials for Cancer

Cancer clinical trial citations were the fifth largest category [288,289,290,291,292,293,294,295,296,297,298,299,300,301,302,303,304,305,306,307,308,309,310,311,312,313,314,315,316,317,318,319,320,321], comprising 9% of total citations (n = 34) (Figure 6E). Although most cancer studies were conducted during recent decades, a minority were D-RCTs (47%) and only half evaluated the effects of enhanced bioavailability products. These studies were of average size (mean, n = 60) and duration (mean = 2.6 months) relative to the other major categories. The number of studies focused on cancer was relatively small, especially when considering the range of diverse cancers studied, including prostate (n = 5) [288,289,290,291,292], breast (n = 5) [293,294,295,296,297], colorectal or its precursors (n = 4) [298,299,300,301], multiple myeloma (n = 4) [302,303,304,305], head and neck (n = 3) [306,307,308], gynecologic (n = 3) [309,310,311], pancreatic (n = 2) [312,313], gastric (n = 1) [314], thyroid (n = 1) [315], and bladder (n = 1) [316], as well as studies evaluating solid tumors of various types (n = 5) [317,318,319,320,321]. Endpoints varied with cancer type and focused on the amelioration of treatment-related side effects, rather than disease progression. For instance, among four D-RCTs examining the effects of curcumin on prostate cancer, one study reported reductions in biomarkers of oxidative stress [290], two studies reported benefits for urinary symptoms, including those secondary to benign prostatic hypertrophy [289,291], and one study did not find any effects on radiation-induced toxicity [292]. The five breast cancer citations reported on disparate endpoints, including the effects of radiation-induced dermatitis (n = 3 [n = 2 D-RCT]) [295,296,297], with the results indicating a lack of improvement in inflammatory biomarkers, pain, or quality of life, and mixed outcomes with respect to dermatitis. The two remaining non-D-RCT breast cancer citations reported an improved response rate to anthracycline-based neoadjuvant chemotherapy (non-D-RCT) and improved quality of life and hematologic parameters during paclitaxane therapy (case series) [293,294]. Colorectal cancer (CRC) citations (n = 4) in diverse populations focused on prevention (no effect), response to chemotherapy or radiation (no effect), or tolerability in metastatic CRC. Solid tumor citations evaluating amelioration of chemotherapy and/or radiation-induced side effects (n = 4) reported positive effects [317,318,320]; body composition was unchanged in a fifth solid tumor study [321]. This latter finding stands in stark contrast to a severe adverse effect of curcumin on body composition documented in a pancreatic cancer trial [313].

### 2.10. Clinical Trials for Less Commonly Studied Disorders

Among the less commonly studied disorders (Other, Figure 4), cardiovascular trial citations (5% of total citations, n = 20) [322,323,324,325,326,327,328,329,330,331,332,333,334,335,336,337,338,339,340,341] primarily focused on the vasculature (n = 11, e.g., compliance and endothelial function) [322,323,324,325,326,327,328,329,330,331,332]. These studies generally reported improvements in subjects who were healthy or had a range of dysfunction, excluding children (here with tetralogy of Fallot [332]), who, it should be noted, were rarely included in curcumin clinical trials. Renal clinical trial citations (3%, n = 13) [359,360,361,362,363,364,365,366,367,368,369,370,371], which examined a range of endpoints and conditions, including contrast-induced nephropathy, diabetic nephropathy, and end-stage renal disease, were too disparate and few in number to discern specific patterns of response. Reproductive organ trial citations (3%, n = 11) [372,373,374,375,376,377,378,379,380,381,382] examined a range of disorders, with polycystic ovarian syndrome (PCOS), a condition associated with insulin resistance [421], being the most common (n = 4) [372,373,374,375,376,377,378,379,380,381,382] where improvements in metabolic function were noted analogous to outcomes reported in other insulin-resistant populations. Pulmonary clinical trial citations (2%, n = 8) [383,384,385,386,387,388,389,390] mostly focused on asthma (n = 5) [384,385,386,387,388], where benefits were reported in all trials, including one focused on children [388]. Dermatological trial citations (2%, n = 7) that focused on inflammatory skin conditions due to autoimmune disorders or external irritants reported benefits (n = 4) [391,392,393,394], while no effects on erythema or barrier function were noted with normal skin (n = 3) [395,396,397]. Miscellaneous other diseases (6%, n = 21) with even fewer citations (e.g., infectious diseases [n = 4] [398,399,400,401], ophthalmologic [n = 4] [402,403,404,405], or hematological disorders [n = 4] [406,407,408,409]) were grouped together, and due to their variability and small numbers cannot be easily summarized [398,399,400,401,402,403,404,405,406,407,408,409,410,411,412,413,414,415,416,417,418]. However, inflammatory or oxidative stress biomarkers were frequent endpoints for these studies, which yielded generally consistent reports of improvement (e.g., decreased inflammatory cytokines in COVID-19 patients [401]).

## 3. Discussion

Humans have used curcumin-containing turmeric (*Curcuma longa* L.) medicinally for thousands of years, primarily for the treatment of inflammatory conditions, as documented by historical medical texts and archeological evidence. While continuous medicinal use for such an extended time period is supportive of the likelihood that curcumin can yield biological effects in humans, this scoping review of curcumin clinical trial outcomes was undertaken to assess scientific evidence querying this postulate, both with respect to diseases treated and biological processes targeted. After a comprehensive search of eight databases for publications, abstracts, and clinicaltrials.gov citations from 1900 to 2020, strong scientific evidence emerged from clinical trials, which primarily (70%) utilized gold standard D-RCT designs, indicating that curcumin can impact disease conditions in humans. Evidence was strongest for highly studied diseases where inflammation, which remains an important etiologic contributor for over 50% of deaths worldwide [422], is an important disease driver. Scientific evidence of anti-inflammatory effects of curcumin in humans demonstrated through clinical and biomarker endpoints in clinical trials for various diseases adds to a long history of evidence stretching back millennia from ethnobotanical use and is consistent with modern molecular evidence of curcumin blockade of key mediators of inflammation [19,20,22,23].

Systematic comparison of botanical studies for scoping or systematic reviews is difficult due to the disparate chemical composition of plant-derived products tested, which is also often not well validated. Additionally, the testing of various enhanced bioavailability curcumin products (45% of all citations), while particularly relevant due to their commercial availability to consumers (55% of turmeric supplements sold in the United States [4]), also makes study comparisons difficult. While direct comparisons by curcumin dose across studies is thus an invalid means of comparison, by limiting our analysis to studies testing the oral administration of curcumin-containing products for disease treatment, where curcumin was the only variable tested, distinct patterns still emerged. This was most notable for the two most frequently studied disorders. For each of several clinical and biomarker endpoints tested, beneficial curcumin effects were noted on average in 79% of trials investigating: (1) metabolic disorders caused by obesity-associated inflammation and insulin resistance [423], which represented 29% of the citations (n = 114) when including polycystic ovary syndrome (PCOS); or (2) osteoarthritis which represented 8% of the citations (n = 31), and is also an inflammatory disorder with obesity as a major risk factor [424]. Despite a minority of citations in these two categories not reporting benefits for certain endpoints, which may be due to factors such as differences in study population, power, endpoints studied, product choice, dosing, or duration, the majority of evidence for these widely studied disease processes (predominantly derived from gold-standard D-RCTs [76–79%] that also included some of the largest studies, thus minimizing bias [425]), supported the health benefits of curcumin for these populations. Thus, consistent with the high prevalence of obesity in the United States (42% of US adults [426]), these findings suggest the possibility that large segments of the US adult population may potentially derive benefits from curcumin use, including adults with: (1) pre-diabetes (38% of US adults [427]) where lifestyle management is key; (2) diabetes (11% [428]); and/or 3) osteoarthritis (11% [429]).

There are of course caveats and limitations associated with any conclusions drawn from scoping reviews assessing the general state of a field, as compared to a systematic review or meta-analysis. However, clear benefits also accrue, as was perhaps most notable here for cancer clinical trial citations. Unlike citations related to metabolic syndrome or musculoskeletal inflammation that comprised half of the available literature and were primarily D-RCTs testing enhanced bioavailability products in larger cohorts, cancer trial citations did not provide a strong evidence base for curcumin use due to low citation numbers and trial designs. Evidence for symptom management was sparse given the small number of citations available for any given tumor type and effects on disease progression were rarely a focus of study. Cancer trial quality, in general, was lower than for other topics (e.g., only 47% D-RCT) with trends over time suggesting that this topic is not a strong focus of current interest. This contrasts with current usage patterns, however, as, for example, 16% of breast cancer survivors in a recent large epidemiologic study reported concurrent curcumin use during chemotherapy despite an absence of data supporting efficacy, or, perhaps even more importantly, safety when used in this context [10]. The serious adverse effects of curcumin reported in one pancreatic cancer trial stand as a cautionary tale when considering use in a cancer population [313].

Secular trends uncovered by this scoping review also yielded interesting findings suggestive of a maturation in certain fields of study. This was most notable for curcumin clinical trials evaluating cognition and memory, where two early negative D-RCTs evaluating non-bioenhanced curcumin in Alzheimer’s disease (AD) populations [247,248] were followed by multiple D-RCT trials with generally positive outcomes focusing on prevention in aging non-AD populations in studies that were higher powered and tested enhanced bioavailability products [239,240,242,243,244,245,246]. A careful review of the entirety of curcumin clinical trial citations also yielded insights that could be missed in more focused disease-specific queries. For example, in curcumin trials focused on diseases affecting the gastrointestinal (GI) mucosa, evidence of benefits was most robust for colonic disorders and weaker for upper GI tract disorders, which may be due to the differential disposition and metabolism of curcumin in the gastrointestinal system, where curcumin is eliminated via the enterohepatic circulation and the microbiome likely impacts its metabolism [2,430]. Another strength of this scoping review was our inclusion of abstracts and other forms of unpublished data, a strategy that can help to mitigate publication bias, which can adversely affect outcomes for both scoping and systematic reviews [26,27].

One limitation of this scoping review is the lack of inclusion of 2021–2022 citations due to pandemic-related delays in data analysis after identification of citations. However, this circumstance allows for comparison of results from this scoping review with those of a systematic review of curcumin clinical trials completed in 2020 [431], which provides both corroborating and additional evidence [431]. The two studies cannot be directly compared since citations in the systematic review were not identified and were fewer in number despite additional inclusion of trials with non-oral curcumin delivery and botanical mixtures, albeit after searching only four databases (vs. eight here) with an earlier end date (mid-2020 vs. end of 2020 here). However, certain comparisons are instructive. Risk of bias assessments in the systematic review exceeded our quality assessment of curcumin clinical trials based on D-RCT design (70% of citations in this scoping review) by inclusion of two additional criteria, incomplete outcome data acknowledgment and selective reporting. The systematic review reported a 48% compliance rate for all parameters in curcumin trials assessed, which increased to 67% in recent years, an encouraging trend consistent with general findings reported here, where many—but not all—studies utilized optimal designs. Additionally, and in contrast with the types of information summarized in the cancer-focused systematic review [431], a particular strength of this scoping review is its summation of curcumin clinical trial outcomes for all diseases using mechanistic groupings (e.g., metabolic disorders associated with obesity), which provides a unique perspective and contribution to the curcumin literature, including citations and search strategies.

Lastly, it is important to note that few clinical trials analyzed in this scoping review examined dose-dependent effects of a single agent, comparative effects of disparate products, and/or provided pharmacokinetic data to facilitate cross-comparison across studies. Thus, best practices for clinical curcumin use, even in conditions where the preponderance of existing evidence supported benefits, remain uncertain and would benefit from the conduct of additional well-funded and carefully designed studies, informed by over three decades of curcumin clinical trial results, as summarized here.

In conclusion, based on the results of this scoping review, curcumin does appear to have biological activity in humans, with significant evidence that curcumin may have medicinal benefits in the treatment of certain inflammatory and/or obesity-related conditions that are common contributors to higher mortality, morbidity, and loss of productivity in the workforce.

## 4. Methods

### 4.1. Design of Systematic Literature Search

A literature review was conducted using recommended five-step scoping review guidelines [26,27]: (1) identify a research question (outcomes and diseases targeted in clinical trials assessing curcuminoid-containing turmeric products); (2) identify relevant studies; (3) select relevant studies; (4) chart data from these studies; and (5) collate, summarize, and report the results. Following reporting guidelines specified in the “PRISMA Extension for Scoping Reviews (PRISMA-ScR,)” [28], a medical librarian (CLH) used both controlled vocabulary terms (e.g., MeSH, Emtree) and keywords to search the following eight databases for clinical studies of curcuminoids in the treatment of medical conditions in humans using database-specific search strategies (Supplemental Figure S1): Ovid/MEDLINE (1966–2020), Cochrane Central (1996–2020), Elsevier/Embase (1947–2020), Clarivate/WOS (1900–2020), EBSCO/CINAHL (1937–2020), EBSCO/PsycInfo (1887–2020), AMED (1985–2020), and ClinicalTrials.gov (1997–2020). Initial searches were completed on 28 May 2019, and updated on 20–21 December 2020. An English language filter was applied; there were no publication date or publication type limits. Additional citations listed within studies were also screened.

### 4.2. Methods for Assessing Citation Inclusion

All identified records were exported to the management software EndNote Version X9 (Clarivate Analytics, Philadelphia, PA, USA), which was used to document and delete duplicate records and pre-screen out animal studies and publications unavailable in English (CLH). Two independent reviewers (TMP, BB) screened the titles and abstracts of all remaining articles for relevance to the topic. Disagreements were resolved by consensus and consultation with the senior author (JLF). The remaining titles and abstracts were then rescreened for relevance, only retaining citations for clinical trials testing oral formulations with study designs allowing for assessment of curcuminoid products as a sole variable (curcuminoid products containing piperine to enhance curcumin bioavailability were retained). Citations reporting different outcomes from a single study were retained, while citations lacking trial data (e.g., study design only) or referencing duplicate data from a single study were excluded.

### 4.3. Data Extraction and Synthesis

All included citations were categorized according to general organ system and further subdivided into disease categories (JFL). All citations for a given organ system were read in their entirety by a single reviewer (TMP, BB, MH, AMR). The following data were extracted and collated from the selected publications: year of publication, disease and population studied, study design, study size and duration, product type and dose, and disease outcomes assessed (clinical and lab-based). Collated data for a given organ system and/or disease were then summarized for report here (TPM, a 4th year medical student; and JLF, an internist and clinical endocrinologist). Limited statistical analyses, consistent with the design and purpose of this scoping review, were conducted using Prism software (GraphPad, San Diego, CA, USA).

## Data Availability

No new data were created in this study; all citations are publicly available.

## Supplementary Materials

The following supporting information can be downloaded at: https://www.mdpi.com/article/10.3390/ijms24054476/s1.
