# Supplementary material for: Curcumin Supplementation and Human Disease: A Scoping Review of Clinical Trials
Source: Int J Mol Sci. 2023 Feb 24;24(5):4476. doi: 10.3390/ijms24054476 (PMC10003109; doi:10.3390/ijms24054476)
Supplement: Supplementary file 1 [file ijms-24-04476-s001.zip › ijms-2227114-supplementary.pdf]

Panknin TM et al,

Curcumin Supplementation and Human Disease: A Scoping Review of Clinical Trials

Supplemental Figure S1: Curcumin Clinical Trial Database Search Strategies

All searches were conducted on May 28, 2019 and updated on December 20-21, 2020. Searches were customized for each database to include appropriate keywords and controlled vocabulary terms related to the use of curcuminoids in the treatment of medical conditions in humans. An English language filter was applied.

Ovid/MEDLINE

#	Searches
1	exp Curcumin/
2	exp Curcuma/
3	"c.longa".ab,kw,ti.
4	"curcum*".ab,kw,ti.
5	Diferuloylmethane.ab,kw,ti.
6	"Nanocurc*".ab,kw,ti.
7	turmeric*.ab,kw,ti.
8	tumeric*.ab,kw,ti.
9	Halada.ab,kw,ti.
10	Haldi.ab,kw,ti.
11	Haridra.ab,kw,ti.
12	"Indian Saffron".ab,kw,ti.
13	Nisha.ab,kw,ti.

14	"Jiang Huang".ab,kw,ti.
15	Rajani.ab,kw,ti.
16	Safran Bourbon.ab,kw,ti.
17	Safran de Batallita.ab,kw,ti.
18	Safran de Indes.ab,kw,ti.
19	"Yu Jin".ab,kw,ti.
20	or/1-19
21	exp controlled clinical trial/
22	randomized controlled trial.pt.
23	controlled clinical trial.pt.
24	"allocat*".ab,kw,ti.
25	"blind*".ab,kw,ti.
26	crossover.ab,kw,ti.
27	"intervention*".ab,kw,ti.
28	"placebo*".ab,kw,ti.
29	"pre post".ab,kw,ti.
30	prospective.ab,kw,ti.
31	"test retest".ab,kw,ti.
32	"trial*".ab,kw,ti.
33	random*.ab,kw,ti.
34	or/21-33
35	20 and 34

36	exp animals/ not exp Humans/
37	35 not 36
38	limit 37 to english language

Cochrane Library

ID	Search
#1	MeSH descriptor: [Curcumin] explode all trees
#2	MeSH descriptor: [Curcuma] explode all trees
#3	curcum*:ti,ab,kw
#4	Diferuloylmethane:ti,ab,kw
#5	c.longa:ti,ab,kw
#6	Nanocurc*:ti,ab,kw
#7	turmeric*:ti,ab,kw
#8	tumeric*:ti,ab,kw
#9	halada:ti,ab,kw
#10	Haldi:ti,ab,kw
#11	Haridra:ti,ab,kw
#12	"Indian Saffron":ti,ab,kw
#13	nisha:ti,ab,kw
#14	"Jiang Huang":ab,kw,ti
#15	Rajani:ti,ab,kw
#16	Safran Bourbon:ti,ab,kw
#17	Safran de Batallita:ti,ab,kw
#18	Safran de Indes:ti,ab,kw
#19	"Yu Jin":ti,ab,kw
#20	{or #1-#19}
#21	MeSH descriptor: [Animals] explode all trees
#22	MeSH descriptor: [Humans] explode all trees
#23	#21 NOT #22
#24	#20 NOT #23
#25	#24 in Trials

Embase

No.	Query
1	'curcumin'/exp
2	'turmeric'/exp
3	'curcuma'/exp

4	#1 OR #2 OR #3
5	curcum*:ti,ab,kw
6	diferuloylmethane:ti,ab,kw
7	c.longa:ti,ab,kw
8	nanocurc*:ti,ab,kw
9	turmeric*:ti,ab,kw
10	tumeric*:ti,ab,kw
11	halada:ti,ab,kw
12	haldi:ti,ab,kw
13	haridra:ti,ab,kw
14	'indian saffron':ti,ab,kw
15	nisha:ti,ab,kw
16	'jiang huang':ab,kw,ti
17	rajani:ti,ab,kw
18	'safran bourbon':ti,ab,kw
19	'safran de indes':ti,ab,kw
20	'yu jin':ti,ab,kw
21	#5 OR #6 OR #7 OR #8 OR #9 OR #10 OR #11 OR #12 OR #13 OR #14 OR #15 OR #16 OR #17 OR #18 OR #19 OR #20
22	#4 OR #21
23	'animal'/exp
24	'human'/exp
25	#23 NOT #24
26	'crossover procedure':de OR 'double-blind procedure':de OR 'randomized controlled trial':de OR 'single-blind procedure':de OR random*:de,ab,ti OR factorial*:de,ab,ti OR crossover*:de,ab,ti OR ((cross NEXT/1 over*):de,ab,ti) OR placebo*:de,ab,ti OR ((doubl* NEAR/1 blind*):de,ab,ti) OR ((singl* NEAR/1 blind*):de,ab,ti) OR assign*:de,ab,ti OR allocat*:de,ab,ti OR volunteer*:de,ab,ti

27	#22 AND #26
28	#27 NOT #25
29	#27 NOT #25 AND [english]/lim

Web of Science

# 17	(#16) *AND* **LANGUAGE:** (English)<br>*Indexes=SCI-EXPANDED, SSCI, A&HCI, CPCI-S, CPCI-SSH, BKCI-S, BKCI-SSH, CCR-EXPANDED, IC Timespan=1900-2019*
# 16	#14 NOT #15<br>*Indexes=SCI-EXPANDED, SSCI, A&HCI, CPCI-S, CPCI-SSH, BKCI-S, BKCI-SSH, CCR-EXPANDED, IC Timespan=1900-2019*
# 15	TS=(animal* NOT human*)<br>*Indexes=SCI-EXPANDED, SSCI, A&HCI, CPCI-S, CPCI-SSH, BKCI-S, BKCI-SSH, CCR-EXPANDED, IC Timespan=1900-2019*
# 14	#13 AND #1<br>*Indexes=SCI-EXPANDED, SSCI, A&HCI, CPCI-S, CPCI-SSH, BKCI-S, BKCI-SSH, CCR-EXPANDED, IC Timespan=1900-2019*
# 13	#12 OR #11 OR #10 OR #9 OR #8 OR #7 OR #6 OR #5 OR #4 OR #3 OR #2<br>*Indexes=SCI-EXPANDED, SSCI, A&HCI, CPCI-S, CPCI-SSH, BKCI-S, BKCI-SSH, CCR-EXPANDED, IC Timespan=1900-2019*
# 12	TS=trial*<br>*Indexes=SCI-EXPANDED, SSCI, A&HCI, CPCI-S, CPCI-SSH, BKCI-S, BKCI-SSH, CCR-EXPANDED, IC Timespan=1900-2019*
# 11	TS="test retest"<br>*Indexes=SCI-EXPANDED, SSCI, A&HCI, CPCI-S, CPCI-SSH, BKCI-S, BKCI-SSH, CCR-EXPANDED, IC Timespan=1900-2019*
# 10	TS=RCT<br>*Indexes=SCI-EXPANDED, SSCI, A&HCI, CPCI-S, CPCI-SSH, BKCI-S, BKCI-SSH, CCR-EXPANDED, IC Timespan=1900-2019*
# 9	TS=random*

	Indexes=SCI-EXPANDED, SSCI, A&HCI, CPCI-S, CPCI-SSH, BKCI-S, BKCI-SSH, CCR-EXPANDED, IC Timespan=1900-2019
# 8	TS=prospective<br>*Indexes=SCI-EXPANDED, SSCI, A&HCI, CPCI-S, CPCI-SSH, BKCI-S, BKCI-SSH, CCR-EXPANDED, IC Timespan=1900-2019*
# 7	TS="pre post"<br>*Indexes=SCI-EXPANDED, SSCI, A&HCI, CPCI-S, CPCI-SSH, BKCI-S, BKCI-SSH, CCR-EXPANDED, IC Timespan=1900-2019*
# 6	TS=placebo*<br>*Indexes=SCI-EXPANDED, SSCI, A&HCI, CPCI-S, CPCI-SSH, BKCI-S, BKCI-SSH, CCR-EXPANDED, IC Timespan=1900-2019*
# 5	TS=intervention*<br>*Indexes=SCI-EXPANDED, SSCI, A&HCI, CPCI-S, CPCI-SSH, BKCI-S, BKCI-SSH, CCR-EXPANDED, IC Timespan=1900-2019*
# 4	TS=crossover<br>*Indexes=SCI-EXPANDED, SSCI, A&HCI, CPCI-S, CPCI-SSH, BKCI-S, BKCI-SSH, CCR-EXPANDED, IC Timespan=1900-2019*
# 3	TS=blind*<br>*Indexes=SCI-EXPANDED, SSCI, A&HCI, CPCI-S, CPCI-SSH, BKCI-S, BKCI-SSH, CCR-EXPANDED, IC Timespan=1900-2019*
# 2	TS=allocat*<br>*Indexes=SCI-EXPANDED, SSCI, A&HCI, CPCI-S, CPCI-SSH, BKCI-S, BKCI-SSH, CCR-EXPANDED, IC Timespan=1900-2019*
# 1	TS=(curcum* OR diferuloylmethane OR c.longa OR nanocurc* OR turmeric* OR tumeric* OR halada OR haldi OR haridra OR “indian saffron” OR nisha OR “jiang huang” OR rajani OR “safran bourbon” OR “safran de indes” OR “yu jin”)<br>*Indexes=SCI-EXPANDED, SSCI, A&HCI, CPCI-S, CPCI-SSH, BKCI-S, BKCI-SSH, CCR-EXPANDED, IC Timespan=1900-2019*

AMED

	Searches

1	exp Curcuma/
2	"curcum*".ti,ab.
3	"c.longa".ti,ab.
4	diferuloylmethane.ti,ab.
5	"nanocurc*".ti,ab.
6	"turmeric*".ti,ab.
7	"tumeric*".ti,ab.
8	halada.ti,ab.
9	haldi.ti,ab.
10	Haridra.ti,ab.
11	"Indian Saffron".ti,ab.
12	Nisha.ti,ab.
13	"Jiang Huang".ti,ab.
14	rajani.ti,ab.
15	Safran Bourbon.ti,ab.
16	Safran de Batallita.ti,ab.
17	Safran de Indes.ti,ab.
18	"Yu Jin".ti,ab.

19	or/1-18
20	exp clinical trials/ or randomized controlled trials/ or comparative study/ or double blind method/ or random allocation/
21	"random*".ti,ab.
22	"trial*".ti,ab.
23	"test retest".ti,ab.
24	prospective.ti,ab.
25	"pre post".ti,ab.
26	"placebo*".ti,ab.
27	"intervention*".ti,ab.
28	crossover.ti,ab.
29	"blind*".ti,ab.
30	"allocat*".ti,ab.
31	RCT.ti,ab.
32	or/20-31
33	19 and 32
34	exp Animals/

35	exp humans/
36	34 not 35
37	33 not 36

CINAHL

#	Query
S25	(S18 AND S22) NOT S19<br><br>Limiters - Language: English
S24	(S18 AND S22) NOT S19
S23	S18 AND S22
S22	S20 OR S21
S21	(MH "Clinical Trials+") OR (MH "Controlled Before-After Studies") OR (MH "Pretest-Posttest Design+")
S20	placebo* OR random* OR "comparative stud*" OR clinical NEAR/3 trial* OR research NEAR/3 design OR evaluat* NEAR/3 stud* OR prospectiv* NEAR/3 stud* OR (singl* OR doubl* OR trebl* OR tripl*) NEAR/3 (blind* OR mask*)
S19	animal* NOT human*
S18	(S1 OR S2 OR S3 OR S4 OR S5 OR S6 OR S7 OR S8 OR S9 OR S10 OR S11 OR S12 OR S13 OR S14 OR S15 OR S16 OR S17)
S17	TI "Yu Jin" OR AB "Yu Jin"
S16	TI Safran de Indes OR AB Safran de Indes
S15	TI Safran de Batallita OR AB Safran de Batallita
S14	TI Safran Bourbon OR AB Safran Bourbon
S13	TI rajani OR AB rajani
S12	TI "Jiang Huang" OR AB "Jiang Huang"

S11	TI Nisha OR AB Nisha
S10	TI "Indian Saffron" OR AB "Indian Saffron"
S9	TI Haridra OR AB Haridra
S8	TI haldi OR AB haldi
S7	TI halada OR AB halada
S6	TI tumeric* OR AB tumeric*
S5	TI turmeric* OR AB turmeric*
S4	TI nanocurc* OR AB nanocurc*
S3	TI diferuloylmethane OR AB diferuloylmethane
S2	TI c.longa OR AB c.longa
S1	TI curcum* OR AB curcum*

PsycInfo

#	Query
S22	S20 NOT S21
S21	animal* NOT human*
S20	S1 AND S19
S19	(S2 OR S3 OR S4 OR S5 OR S6 OR S7 OR S8 OR S9 OR S10 OR S11 OR S12 OR S13 OR S14 OR S15 OR S16 OR S17 OR S18)
S18	TI "Yu Jin" OR AB "Yu Jin"
S17	TI Safran de Indes OR AB Safran de Indes
S16	TI Safran de Batallita OR AB Safran de Batallita
S15	TI Safran Bourbon OR AB Safran Bourbon
S14	TI rajani OR AB rajani

S13	TI "Jiang Huang" OR AB "Jiang Huang"
S12	TI Nisha OR AB Nisha
S11	TI "Indian Saffron" OR AB "Indian Saffron"
S10	TI Haridra OR AB Haridra
S9	TI haldi OR AB haldi
S8	TI halada OR AB halada
S7	TI tumeric* OR AB tumeric*
S6	TI turmeric* OR AB turmeric*
S5	TI nanocurc* OR AB nanocurc*
S4	TI diferuloylmethane OR AB diferuloylmethane
S3	TI c.longa OR AB c.longa
S2	TI curcum* OR AB curcum*
S1	SU.EXACT("Treatment Effectiveness Evaluation") OR SU.EXACT.EXPLODE("Treatment Outcomes") OR SU.EXACT("Placebo") OR SU.EXACT("Followup Studies") OR placebo* OR random* OR "comparative stud*" OR clinical NEAR/3 trial* OR research NEAR/3 design OR evaluat* NEAR/3 stud* OR prospectiv* NEAR/3 stud* OR (singl* OR doubl* OR trebl* OR tripl*) NEAR/3 (blind* OR mask*)

ClinicalTrials.gov

**curcumin OR turmeric | Studies With Results**
